# MicroRNAs, Stem Cells in Bipolar Disorder, and Lithium Therapeutic Approach

**DOI:** 10.3390/ijms231810489

**Published:** 2022-09-10

**Authors:** Donatella Coradduzza, Giuseppe Garroni, Antonella Congiargiu, Francesca Balzano, Sara Cruciani, Stefania Sedda, Alessandra Nivoli, Margherita Maioli

**Affiliations:** 1 Department of Biomedical Sciences, University of Sassari, 07100 Sassari, Italy; 2 Department of Medical, Surgical and Experimental Sciences, University of Sassari, 07100 Sassari, Italy; 3 Center for Developmental Biology and Reprogramming (CEDEBIOR), Department of Biomedical Sciences, University of Sassari, Viale San Pietro 43/B, 07100 Sassari, Italy

**Keywords:** microRNA, stem cells, neural stem cells, bipolar disorder (BD), lithium, molecular mechanisms

## Abstract

Bipolar disorder (BD) is a severe, chronic, and disabling neuropsychiatric disorder characterized by recurrent mood disturbances (mania/hypomania and depression, with or without mixed features) and a constellation of cognitive, psychomotor, autonomic, and endocrine abnormalities. The etiology of BD is multifactorial, including both biological and epigenetic factors. Recently, microRNAs (miRNAs), a class of epigenetic regulators of gene expression playing a central role in brain development and plasticity, have been related to several neuropsychiatric disorders, including BD. Moreover, an alteration in the number/distribution and differentiation potential of neural stem cells has also been described, significantly affecting brain homeostasis and neuroplasticity. This review aimed to evaluate the most reliable scientific evidence on miRNAs as biomarkers for the diagnosis of BD and assess their implications in response to mood stabilizers, such as lithium. Neural stem cell distribution, regulation, and dysfunction in the etiology of BD are also dissected.

## 1. Introduction

Bipolar disorder (BD) is a severe, chronic, and disabling neuropsychiatric disorder. An estimated lifetime prevalence of 1% and rates reaching as high as 4–5% have been reported for bipolar spectrum disorders [1]. BD is characterized by recurrent episodes of mania or hypomania and depression, with or without mixed features, and subsyndromal symptoms are common [2]. The pathogenesis of BD is not well understood; nevertheless, research is focusing on genetics, neurobiological, and psychosocial factors contributing to the development of the disorder [3], with heritability being ∼70% [1,2,3,4]. The analysis of genetic variation associated with BD susceptibility confirmed its highly polygenic nature, describing many thousands of common variants that are associated, at different levels, with the risk of BD [5]. The diagnosis of bipolar disorder is often subjective due to the complex spectrum of symptoms. From a clinical point of view, the differential diagnosis of BD from other diseases showing overlapping symptoms is still a challenge, thus [5,6] affecting the suitable treatment from the early stages [7]. Within this context, patients could exhibit hypomanic episodes as a mood status, while seeking medical help during depressive episodes [8]. The diagnostic error is approximately 40% and could require 5–12 years to be corrected, leading to a prolonged course, more affective episodes, and an increased suicide rate and socioeconomic burden [9,10]. Within this context, the identification of biomarkers that may reflect BD-specific pathophysiological processes can be helpful to enrich current diagnostic algorithms, thus providing new biological targets for the development of personalized treatments [11,12]. The nervous system (NS) comprises different cell types, including stem cells, that reside in specific niches, capable of generating new neurons, astrocytes, and oligodendrocytes [13]. Different studies highlighted the potential role of miRNAs as promising biomarkers, easily collected from different biological fluids [14,15,16], to reveal the presence and the evolution of BD and drug responses [17]. Different environmental factors or epigenetic modifications involving miRNA could trigger psychiatric disorders. Such as BD, affecting adult neurogenesis by influencing neural stem cell behavior [18,19,20,21]. MiRNAs are responsible for NS development and neurogenesis, proliferation, differentiation, and apoptosis [22,23], being implicated in translation, RNA metabolism, gene development, and regulation [24,25]. MicroRNAs (miRNAs) are a class of small, well-conserved noncoding RNAs that, when dysregulated, could be involved in different nervous system diseases (NSDs) [26,27,28,29,30,31]. Moreover, they are related to the glucocorticoid regulation of the hypothalamic–pituitary–adrenal (HPA) axis in response to stress in mood and anxiety disorders [32,33,34]. MiRNAs are actively secreted by cells either shuttled via microvesicles as exosomes, in complexes with RNA-binding proteins or lipoproteins, such as nucleophosmin (NPM1), or Argonaute proteins [35,36,37]. Circulating miRNAs can be useful candidates for the diagnosis of neuropsychiatric disorders [38,39], being easily detectable in plasma [33]. This review focuses on the role of circulating miR-144, miR-134, and miR-34 as potential biomarkers that are also involved in mood stabilizer treatment of BD [40].

## 2. Stem Cells Epigenetics and BD

Stem cells are involved in maintaining the homeostasis of the whole organism for their extensive ability to self-renew and differentiate into tissue-specific elements. They exert a crucial role in regenerative medicine, being able to acquire a specific phenotype upon stimulation with different chemical or physical stimuli. Moreover, they can be isolated from several available sources [41,42,43,44,45].

The existence of a subset of stem cells in the central nervous system (CNS) able to generate new neurons, astrocytes, and oligodendrocytes has been largely demonstrated [13].

These cells reside in specific regions of the brain and are responsible for the generation of new neurons, astrocytes, and oligodendrocytes [45,46]. Neural stem cells (NSCs) are undifferentiated neural cells with replicative potential, capable of differentiating into multiple neuronal and glial cell types. They retain a quiescent state for long periods, thus providing a pool of reserve cells useful for tissue regeneration [47].

These cells reside in specific niches, such as the subventricular zone (SVZ) of the lateral ventriculus, and the dentate gyrus of the hippocampus in the subgranular zone (SGZ) [48]. The niche mediates the interaction between stem cells and the environment, being able to influence cell fate and behavior. Moreover, in the niche cell–cell interactions, various microenvironmental signals (among which are growth factors and neurotransmitters) act to regulate stem cell quiescence and proliferation, and promote neurogenesis [49,50].

Epigenetics, a physiological process with phenotypic alterations triggered by environmental factors not involving changes in the DNA sequence, could play a key role in the pathophysiology of bipolar disorder (BD). Within this context, microRNAs (miRNAs) may represent an interesting diagnostic tool in neurogenesis and neuro-psychiatric disorders [51,52,53,54].

Different environmental factors, such as stress, exercise, and antidepressant drugs or neurodegenerative disorders, may affect neurogenesis [55].

Epigenetic modification could be involved in adult neurogenesis by influencing stem cells located in the niche [56] by inducing chromatid modifications and thus altering the expression of selected targets, ultimately affecting the maintenance of the proliferation and stemness of NSCs [57].

## 3. MicroRNA Expression in Bipolar Disorder

Emerging omics technologies are providing innovative tools for medical decision-making. Many authors suggest that, among transcriptomic biomarkers, microRNAs (miRNAs) are the most promising. MiRNAs, detectable in liquid biopsy, are highly stable, have a long half-life, their analysis does not require any special handling, and they are quantified with relatively low cost, high sensitivity, and high specificity through standard techniques [58,59], as shown in Table 1.

miRNA expression has been analyzed in different samples, such as post-mortem brain tissue, cerebral spinal fluid (CSF), and peripheral blood from psychiatric patients. The results show dysregulation in some classes of miRNAs in patients with mood disorders and psychosis [60,61,62,63,64,65,66,67,68,69], and with major depression [70,71,72], suggesting a common dysregulation of these miRNAs in different neuropsychiatric conditions [22,73,74,75,76,77,78]. Some authors described lower circulating miR-134 expression in the plasma of bipolar (type I) patients without medication during a manic episode as compared with controls. Strazisar and co-workers (2015) provided strong support for the involvement of miRNAs in the pathogenesis of BD by analyzing 13 miRNA sequences in patients with bipolar disorder and schizophrenia, and healthy controls [79]. A more recent study on peripheral blood samples [80] showed that a number of miRNAs were significantly dysregulated in BD, either overexpressed (miR-720-5p, miR-3158-3p, miR-4521-5p, miR-345-5p, miR-1973-5p, miR-140-3p, miR-30d-5p, miR-330-3p, miR-330-5p, miR-378a-5p, miR-21-3p, and miR-29c-5p) or downregulated (miR-1972-5p, miR-4440-5p, and miR-1915-5p). The same authors hypothesized an implication of long-term potentiation, phosphatidylinositol signaling system, neurotrophin signaling, and gap junction signaling in the pathogenesis of BD [81]. Banach et al. (2017) analyzed the expression level of miR-499, miR-708, and miR-1908 in the leukocytes of bipolar patients during a depressive episode as compared with a remission state. Significant down-regulation of these miRNAs was detected in patients during a depressive state, providing further insights into the pathophysiology of depression in bipolar disorders [82].

### 3.1. MiR-144-5p

**Figure 1:**
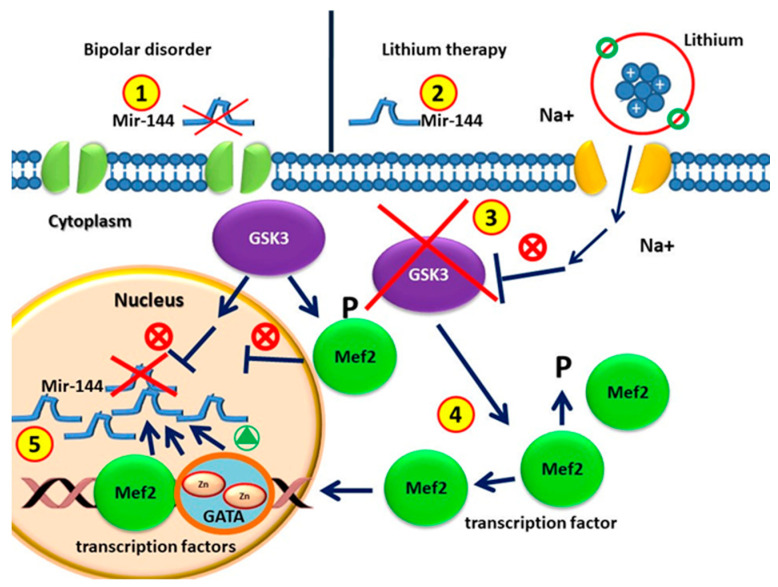
(1) Circulating MiR-144 in patients without drugs with bipolar disorders is lower than that in healthy individuals. (2) MiRNA-144 levels increase after lithium treatment. (3) Glycogen synthase kinase 3 (GSK-3) is the therapeutic target of lithium for BD treatment. (4) GSK3β is involved in regulating gene expression by phosphorylation and therefore destabilizing MEF2. GSK3β activity in BD represses the MEF2 transactivation properties. GSK3β pharmacological inhibition with lithium causes increased activity of MEF2. (5) MEF2 proteins are recruited to the target GATA promoter. GATA transcription factor and MEF2 induce miR-144/451 transcription.

MiR-144 is a family of microRNAs found in mammals, including humans. In humans, miR-144 has been characterized as a “common miRNA signature” [83] of several different tumors. The gene encoding miR-144 is located on chromosome 11 and encompasses a non-coding transcriptional unit comprising miR-451 [84]. The carboxy terminus of the GATA4 transcription factor is thought to activate the transcription of miR-144 [85,86]. Sureban et al. suggested that DCAMKL-1, a microtubule-associated kinase expressed in postmitotic neurons, negatively regulates miR-144 and Notch-1, being itself one of the downstream targets of miR-144 [87]. The miR-144/451 gene is a direct transcriptional target of GATA-1 [88], being itself a direct target of the same hematopoietic transcription factor. Within this context, ChIP studies demonstrated that GATA-1 binds the miR 144/451 locus at the promoter and an upstream enhancer at −2.8 kb, displacing GATA-2 and recruiting the cofactor FOG-1 [88,89]. The myocyte enhancer factor 2 (MEF2) family of human transcription factors are implicated in different cellular and tissue functions, and are also involved in several diseases, such as neurological disorders. MEF2 proteins are co-expressed with members of the GATA family in several other types of cells. The most notable is the presence of MEF2 proteins with GATA-6 in non-muscle cells, including those in the brain [88,89,90] (Figure 1). Given the co-expression of MEF2 and GATA factors, the GATA-dependent MEF2 pathway could also be enrolled to understand the molecular dysregulation occurring during BD.

In a study involving 16 primary health care centers, the plasma miR-144-5p levels were associated with depressive symptoms, being significantly higher after psychological treatment (structured mindfulness-based group therapy) [91]. These results, even if the study did not focus on BD patients, suggest that miRNA-144-5p can reflect one of the pathological processes of depressive symptomatology [90]. Within this context, the signaling pathways targeted by miR-144 include the protein kinase C (PKC), Wnt/β-catenin, and PTEN pathways, all implicated in the development of depression [91]. Moreover, it has recently been demonstrated that MiR-144 can inhibit the expression of Ataxin 1 (ATXN1) in human cells, which in turn is associated with mental disorders, such as bipolar disorder, schizophrenia, and major depressive disorder [91].

### 3.2. MiR-134

**Figure 2:**
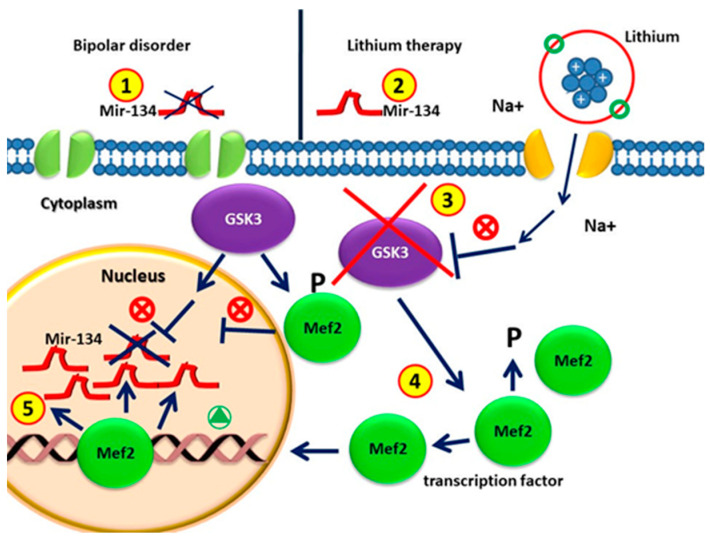
(1) Circulating MiR-134 in patients with bipolar disorders without drugs is lower than that in healthy individuals; (2) MiRNA-134 levels increase significantly after four weeks of lithium treatment; (3) GSK3β is the therapeutic target of lithium in the treatment of BD. (4) GSK3β is involved in regulating gene expression by phosphorylating and thus destabilizing MEF2. GSK3β activity in BD represses the MEF2 transactivation properties. GSK3β pharmacological inhibition by lithium causes increased activity of MEF2. (5) The MEF2 transcription factor leads to the up-regulation of mir-134 in patients with lithium treatment.

Affective disorders are associated with reduced size of brain regions related to mood, cognition, and neuronal atrophy, together with a synaptic loss [92]. Within this context, miRNA-134 is specifically expressed in the brain, being able to counteract dendritic spine formation in vitro [93]. Usually, miR-134 binds to the mRNA encoding the LIMK1 protein kinase, a regulator of synaptic morphogenesis [23,90,94,95]. Moreover, the NAD-dependent deacetylase sirtuin1 also plays an important role (SIRT1) in normal brain physiology and neurological disorders [92]. Within this networking, sirtuin1 (SIRT1) is able to regulate miR-134-5p, as the knockdown of SIRT1 in vitro increases miR-134-5p expression, which in turn inhibits cAMP response element binding (CREB) protein expression [95]. Furthermore, miR-134-5p is also regulated by Mef2, which negatively regulates the number of excitatory synapses in mature hippocampal neurons [96]. The inhibition of Mef2 in rat hippocampal neurons followed by depolarization downregulates miR-134-5p, affecting its ability to regulate its targets, such as CREB. The downregulation of Mef2 greatly reduces the stimulation-induced expression of mir-134 and of the miR-379–410 miRNAs located in the same locus [64,95,96]. Fan et al. showed that the overexpression of miR-134 was sufficient to produce depressive-like behavior. In particular, different authors demonstrated that, in BD patients without medication, the circulating plasma levels of miR-134 were significantly decreased as compared with those of control patients and patients undergoing drug treatment [97]. Following these observations, different authors claimed that decreased plasma miR-134 levels may be directly associated with the pathophysiology and severity of manic symptoms in BD, and may be considered a potential peripheral marker of mania, associated with effective mood stabilizer treatment [97]. MiR-134 could exert an effect on the neurogenesis of neural progenitor cells by inhibiting the GSK3β-Snail path. GSK3 is involved in different pathways of neuronal development and functioning, neuronal morphology, synapse formation, and neurotransmitter signaling, as well as in the onset of several neurological disorders (targeting GSK3 signaling as a potential therapy for neurodegenerative diseases and aging) [98]. It is well known that GSK3 is increased in BD due to a serotonergic hypofunctionality (serotonin syndrome of depression) [99,100], thus representing one of the therapeutic targets of lithium. GSK3β is a negative regulator of MEF2 transcriptional activity in skeletal and cardiac muscle. This effect is indirectly mediated by repressing the p38MAPK path, a well-known positive regulator of MEF2 activity. A variety of loss of function approaches revealed that the abrogation of GSK3β signaling leads to greater transcriptional activity of MEF2, both in vitro and in vivo, in myoblasts and cardiac myocardium. In addition, GSK3β inhibition increases p38MAPK phosphorylation, which in turn increases MEF2 [63,99]. Here, we speculate that the increase in mir-134 in BD patients after treatment with lithium is due to the following mechanism: lithium inhibits GSK3, which increases Mef2, which is able to induce the expression of mir-134 [23,95,101,102] (Figure 2).

### 3.3. MiR-34a

MiR-34a is a regulator of central nervous system (CNS) plasticity. Hippocampus-related neurogenesis is a neural stem cell-based process that is important for several aspects of cognitive abilities [103], also showing a potential therapeutic function by responding to local neuronal loss in pathological events [104]. Within this context, it is noteworthy that the neurogenic potential of NSCs in the adult Dentate Gyrus (DG) decreases throughout life [105].

MiR-34a is an intergenic miRNA located on human chromosome 1 between the coding regions for G Protein-Coupled Receptor 157 (GPR157). In vivo, miR-34a imposes a negative control on the metabotropic glutamate receptor 7 (GRM7) levels in primary hippocampal culture. Glutamatergic neurotransmission is involved in many neuropathological conditions associated with neuropsychiatric disorders. Furthermore, the anti-miR inhibitor miRNA-34a significantly increases the GRM7 protein levels, demonstrating that miR-34a and GRM7 coexist, at least in cultured hippocampal neurons, and that endogenous miR-34a is sufficient to control the GRM7 protein levels [106,107].

**Figure 3:**
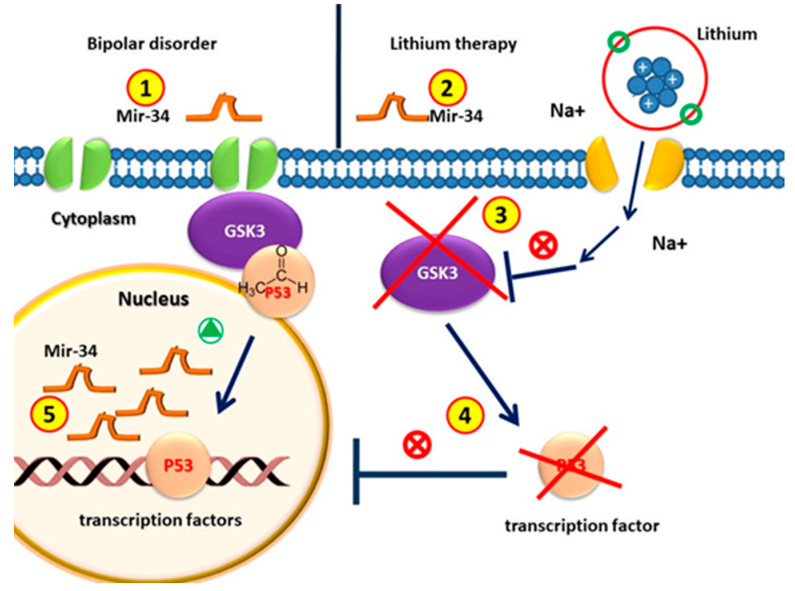
Circulating MiR-34 in patients with bipolar disorder is higher than that in healthy individuals. (2) MiRNA-34 levels decrease after lithium treatment. (3) GSK3β is the therapeutic target of lithium in the treatment of BD. (4) GSK3β is involved in the regulation of gene expression by phosphorylating p53; the pharmacological inhibition of GSK3β with lithium counteracts p53 activity. (5) Since p53 induces the transcription of miR-34, without p53, the amount of MiR-34 in patients with lithium treatment decreases.

These results, showing that miR-34a is down-regulated, lead to the predicted up-regulation of its effector GRM7 in both in vivo and cultured hippocampal neurons by lithium and VPA (Figure 3).

Several studies show that downregulated miR-34a is neuroprotective after combination with Li treatment, whereas the overexpression of miR-34a induces neuronal death in human SH-SY5Y cells [108]. The expression levels of mir-34a in the prefrontal cortex were lower in subjects with BD as compared with controls [60,68,109].

**Table 1:** MicroRNA expression studies in bipolar disorder.

miRNAs	Samples	Method	Expression	Gene Function Pathway	References
miR-17-5p, miR-579, miR-106b-5p, miR-29c-3p miR-145-5p, miR-485-5p, miR-370, miR-500a-5p, miR-34a-5p, and miR-508-3p	Human pre-frontal cortex 15 BD * 15 SCZ * 15 MD * 15 CTRL *	TDLA array	BD * vs CTRL * Overexpressed in BD * Underexpressed in BD * Shared miRNAs alteration with SCZ * and MD *		Smalheiser et al., 2014 [68]
miR-34a	Human ACC–LCH 89 BD * 96 CTRL	TaqMan qRT-PCR	Overexpressed in BD *	Neuronal differentiation, synaptic protein expression, and neuronal	Bavamian et al., 2015 [109]
miR-34a	Human ACC * 8 BD * 14 CTRL *	PCR based TDLA	Underexpressed in BD *	NCOA1 and NCOR2: modulation of transcriptional activity at the glutamatergic rec. PDE4B: regulation of cAMP signaling in synapses	Azevedo et al. (2016) [60]
miR-720-5p, miR-3158-3p, miR-4521-5p, miR-345-5p, miR-1973-5p, miR-140-3p, miR-30d-5p, miR-330-3p, miR-330-5p, miR-378a-5p, miR-21-3p, miR-29c-5p, miR-1972-5p, miR-4440-5p, and miR-1915-5p	Human peripheral blood 20 BD * 20 MDD * 20 CTRL *	TaqMan qRT-PCR	Overexpressed in BD * Underexpressed in BD *	Long-term potentiation, phosphatidylinositol signaling system, neurotrophin signaling, and gap junction	Maffioletti et al., 2016 [80]
miR-504, miR-145, miR-22, miR-133b, miR-154, miR-889, miR-454, miR-29a, miR-767-5p, miR-874, miR-32, and miR-573	Human brain tissue (PFC) * 35 BD * 35 SCZ * CTRL *	TDLA array	BD vs. CTRL Overexpression Underexpression	Negative correlation between the expression of miRNAs and their predicted gene targets: TH*, PGD *, GRM3 *(neurodevelopment and behavior pathways)	Kim et al., 2016 [62]
miR-499, miR-708, and miR-1908	Human peripheral blood (leukocytes) 15 BP 17 MDD *	TaqMan qRT-PCR	BD *-depressive vs. BD *-remission state: Downregulated		Banach et al. (2017) [82]

* Abbreviations: bipolar disorder (BD), control (CTRL), schizophrenia (SCZ), major depressive disorder (MD), phosphogluconate dehydrogenase (PGD), metabotropic glutamate receptor 3 (GRM3), thyroid hormone (TH), major depressive disorder (MDD), prefrontal cortex (PFC), lissencephaly with cerebellar hypoplasia (LCH), anterior cingulate cortex (ACC).

## 4. MicroRNAs, Stem Cells, and Response to Mood Stabilizers in Bipolar Disorder

Several studies focused on miRNA expression after treatment, particularly in response to mood stabilizers, as shown in Table 2. The first pre-clinical study in non-human (rats) showed miRNA dysregulation in the hippocampus after chronic treatment with mood stabilizers (MD), lithium, or valproate. Under-expression was proven for let-7b, let-7c, miR-128a, miR-24a, miR-30c, miR-34a, and miR-221 while over-expression was found for miR-144 [106]. In the same year, Chen and collaborators (2009) published results from another pre-clinical study that aimed to evaluate miRNA expression after treatment with lithium in lymphoblastoid cell lines. In particular, after 4 days of treatment, they observed the over-expression of miR-221, miR-152, miR-15a, miR-155, miR-34a, and miR-181c, and under-expression of miR-49. After 16 days of treatment, only the over-expression of miR-34a, miR-152, miR-155, and miR-221 was detected. The authors hypothesized that Li may have induced miRNA expression changes, leading to post-transcriptional regulation, or it may have regulated the expression of genes involved in the transcription machinery, including miRNAs. The important clinical implications following these findings are that miRNAs responding to Li treatment may serve as biomarkers to understand different treatment outcomes in individual patients [40]. In a more recent pre-clinical study aimed at evaluating miRNA expression changes following glutamate-induced excitotoxicity in cerebellar granule cell cultures (CGCs) of rats and neuroprotective treatment with lithium and valproate, after treatment, miR-34a and miR-495 were significantly down-regulated while miR-182, miR-147, and miR-222 were over-expressed. They concluded that the pathways associated with mood-stabilizer-regulated miRNAs were strongly associated with the pathways implicated in both neuropsychiatric and neurodegenerative disorders [108].

Lithium (Li) is considered a key treatment for the acute and long-term management of BD [7], but responses to treatment can be considerably different; indeed, only one-third of patients treated with lithium are responders. Several clinical and biological factors are linked to a favorable prophylactic response. The modulation of gene function by Li has been suggested as a plausible mechanism, although the molecular mechanisms are not well understood [106]. Li produces direct interactions on several regulatory sites, including lithium-sensitive magnesium-dependent phosphatases (IMPase) and glycogen synthase kinase 3 beta (GSK3β). GSK3β is a negative regulator of MEF2 transcriptional activity. MEF2 proteins are recruited to target promoters by GATA transcription factors, thus enhancing their transcriptional activity. Li also modulates enzymes involved in the production of GABA, dopamine, and glutamate (NMDA receptors), and influencing different signaling systems, such as the ERK/MAPK, PKC, PI3K/Akt, and Wnt/b-catenin pathways.

Within this context, Li is able to improve the dysregulated mitochondrial function found in IPSCs derived from BD patients [110,111].

Moreover, neuronal processes, such as neurite outgrowth, neurogenesis, remodeling of neuronal structure and neuroprotection (BDNF and GDNF), and angiogenesis (VEGF), are modulated by Li treatment. It is also noteworthy that ketamine, a drug with a rapid anti-depressive effect, is involved in molecular neuroplasticity. It has also been hypothesized that this drug may act through the regulation of miRNAs [112]. Some of these miRNAs are critical modulators of central nervous system (CNS) plasticity. Among these, miR-34 contributes to and regulates the effects of lithium on the metabotropic glutamate receptor, GRM7, both in vitro and in vivo [22,106,108]. Lithium-treated cells showed an increase in the anti-apoptotic protein BCL2 and a decrease in the expression of the pro-apoptotic protein BAX. Li is also able to reduce the production of reactive oxygen species activating the redox-sensitive transcription factor NRF2, thus increasing the expression of its target genes. In contrast, NRF2 knockdown reduces the neuroprotective, anti-apoptotic, and antioxidant effects of lithium [113].

### 4.1. MiR-144

MiR-144 is widely expressed in the brain. Several studies demonstrated that miR-144 is involved in the response to mood stabilizer treatment [106], in stress responses [33,114], and in diseases related to aging [115]. Long-term treatment with lithium results in a significant increase in miR-144 expression in the rat hippocampus, a region involved in mood regulation [106]. In humans, miR-144 acts on the protein kinase C (PKC), Wnt/β-catenin, and PTEN pathways. This miRNA can also inhibit the expression of Ataxin 1 (ATXN1), a gene associated with bipolar disorder, schizophrenia, and major depressive disorder. Therefore, it has been identified as one of the potential targets for the development of new antipsychotic drugs for the treatment of schizophrenia and bipolar affective disorder [14], and for the development of mood stabilizers [53]. Figure 1 shows the biological mechanism.

### 4.2. MiR-134

It is well known that the miRNA-134 plasma levels begin to increase after 2 weeks, being significant after 4 weeks of lithium treatment in bipolar patients as compared with untreated BD patients. Changes in the miR-134 blood levels are associated with the recovery of secondary manic symptoms due to long-term treatment with mood stabilizers. Furthermore, miR-134 is involved in the development of dendritic spines blocking the expression of protein kinase, Limk1, which controls synaptic development, maturation, and/or plasticity [23]. Within this network, the brain-derived neurotrophic factor (BDNF), a neurotrophin released in response to synaptic stimulation, is able to release the inhibition of Limk1 translation imposed by miR-134 [23]. Therefore, it appears that miRNA-134 and BDNF have opposite actions in the regulation of dendritic spine development. Increasing the BDNF levels after treatment with mood stabilizers appears to inhibit Limk1–miR-134 mRNA binding. The authors hypothesized that this interaction may be associated with the increased plasma levels of miRNA-134 (Figure 2).

### 4.3. MiR-34a

Several studies demonstrated that miR-34a is downregulated following Li neuroprotective treatment. This miRNA is neuroprotective in primary rat neuronal cultures, in human SH-SY5Y cells [91] in the hippocampus [106], and in rodent cerebellar granule cell cultures [78]. In vitro, the overexpression of miR-34a significantly reduces the mRNA levels of BCL-2, NRF2, and BDNF in SH-SY5Y cells by inducing neuronal death, while GRM7 levels [60,93,106,108,109,113,116,117] are induced upon miR-34a downregulation. Currently, the mechanisms of the effect of lithium on miR-34a expression are still unknown; nevertheless, different transcription factors, signaling pathways, and epigenetic changes might be involved [41,118]. MiR-34a appears to be the link between the response to therapeutic treatment and the molecular mechanisms [109]. MiR-34a, in non-neuronal cells, has been shown to be a strong inhibitor of Wnt signaling and β-catenin-mediated transcription in response to p53 activation [87]. Recent findings have shown that Actin/spectrin adaptor protein (ANK3) positively regulates both the cadherin and Wnt signaling pathways, altering β-catenin availability; loss of ANK3 function impacts neurogenesis [109], supporting the importance of Wnt signaling in the etiology of neurodevelopmental disorders. Since lithium also affects Wnt signaling, it could be hypothesized that high expression of miR-34a may be important for the pathophysiology of BD via this pathway. It is believed that the delayed therapeutic effects of mood stabilizers may be partly attributed to their ability to reduce the miR-34a levels following chronic treatment [87]. Using IPA analysis, Husenbeger et al. (2013) identified TGF-β signaling associated with miR-34a. TGF-β expression has been implicated in several brain injuries (cerebral ischemia, traumatic brain injury, and AD) [95] and in the dorsolateral prefrontal cortex of patients with schizophrenia [96]. TGF-β has also been shown to provide neuronal protection against excitotoxic injury and, in cortical neurons, TGF-β signaling is regulated by lithium through the inhibition of GSK-3 [100]. This suggests that the neuroprotective effects of Li treatment may modulate TGF-β signaling via the regulation of miRNA-34th in order to protect against glutamate insult [108].

## 5. Neural Stem Cells

In adulthood, new functional neurons can be generated from stem cells, a phenomenon known as adult neurogenesis (AN). In animal models, reduced AN has been implicated in the etiopathology of psychiatric disorders, such as bipolar disorder [21].

Lithium has been used in modern psychiatry for the long-term treatment of bipolar disorder, being also able to exert hematological, antiviral, and neuroprotective effects [119]. Lithium exerts an effect on neurogenesis by increasing the proliferation of progenitor cells in the dentate gyrus of the hippocampus and increasing the mitotic activity of Schwann cells [119].

Within this context, some authors reported that Li shows neuroprotective and neurotrophic properties, which may be related to its clinical effectiveness [120].

In addition, therapeutic concentrations of Li have recently been shown to significantly increase neuronal proliferation and the differentiation of neural progenitor cells in vitro [121,122].

It has been demonstrated that Li could promote NSC proliferation by activating the Wnt signaling pathway in vitro. This effect provides an excellent target for the development of new treatments for those diseases involving NSC dysfunction [123].

Among the different factors able to exert an effect on neural stem cells, miRNAs play an important role both in neural development and in the adult brain, being dysregulated in neuropsychiatric disorders, such as BD [124], as demonstrated by postmortem brain tissue isolated from bipolar patients [125].

Within this context, some authors proposed that miR-34a is implicated in multiple etiological factors and the pathogenesis of bipolar disorder, along with neuronal development and synaptogenesis.

Using human neuronal progenitor cells derived from iPSCs, they showed that an increase in miR-34a expression impairs neuronal differentiation [109].

Neuronal differentiation and cell morphology, synapse function, and electrophysiological maturation are significantly impaired in NSCs upon miR-34 overexpression, which is usually faintly expressed during physiological neurogenesis [126].

The overexpression of miR-34a results in increased postmitotic neurons and the elongation of neurites in mouse NSCs. A decrease in its expression resulted in the inhibition of neuronal differentiation. MiR-34a would induce neuronal differentiation by targeting the Notch ligand Delta-like 1 (Dll1) gene, negatively regulating cell proliferation [127,128].

MiR-34a blocks proliferating PC12 cells in G1 stages, a pre-requisite for neuronal differentiation, and is upregulated in aged brain cells, suggesting that increased expression of miR-34 is necessary to maintain mature neurons in the non-proliferative stage [129].

Another miRNA related to neurogenesis and bipolar disorder is mir-134. The miR-134 plasma levels are downregulated in patients with BD without medication, being associated with the pathophysiology and severity of manic symptoms in BD, since, after drug treatment, the levels increase [93].

In particular, in the central nervous system, miR-134 plays an essential role in embryonic stem cell proliferation and differentiation by suppressing Nanog and inducing neural development. Moreover, the upregulation of this miRNA was described during oligodendrogliomas (ODG) and glioblastomas (GBM), suggesting its possible involvement in brain tumor progression [130].

MiR-134 is also able to influence the regulation of dendritic spine volume and synapse formation in mature rat hippocampal neurons in vitro, acting on LimK1 protein kinase [23]. The role of miR-134 in neuroplasticity is also inferred by its capability to promote neural progenitor proliferation, while counteracting Chrdl-1-induced apoptosis and Dcx-induced differentiation in vitro [131].

## 6. Clinical Implications and Limitations

BD results from the complex interaction of multiple susceptible genes with environmental factors, resulting in not only mood disorder, but also a constellation of cognitive, motor, autonomic, endocrine, and sleep/wake abnormalities. The discovery of the role of miRNAs in diagnosis, prognosis, and drug response/resistance may improve clinical approaches and prognosis in psychiatric patients based on current concepts of personalized medicine applied to mental disorders, ‘precision psychiatry’ [132,133]. MiRNA expression, also induced by Li treatment, leads to post-transcriptional regulation. Li itself has been shown to regulate the expression of genes related to BD, including miRNAs. Finally, the selected miRNAs targeted by Li treatment can serve as biomarkers to understand different treatment outcomes in different patients. Identifying target miRNAs and their regulated transcripts may be important to further unravel the mechanism of action of Li and disclose a novel future role of neural stem cells in brain homeostasis as a promising tool for stem-cell-based therapy and as a promising possible therapeutic tool suitable for BD therapy.

## 7. Rationale behind Article Selection

The literature included in this review was identified through MEDLINE/PubMed/Index Medicus, PsycINFO/PsycLIT, Excerpta Medica/EMBASE, the Science Citation Index at Web of Science (ISI), and the Cochrane library databases. The reference terms used for the search were “circulating miRNAs”, “psychiatric disorders and mood stabilizer treatment”, “miRNA and bipolar disorder”, “bipolar”, “manic-depressive”, “Lithium”, “valproate”, “valproic acid”, “Neural stem cells and bipolar disorder, “Neural stem cells and miRNA 34-a”, “Neural stem cells and miRNA 134”, “Neural stem cells and miRNA 144-5p”, “Neural stem cells and lithium”, and their combinations. Data from expression studies (both post-mortem and circulating) performed in bipolar patients were considered, including studies on patients being treated with lithium. Studies focusing exclusively on other psychiatric disorders, such as schizophrenia or major depression, were excluded. The data incorporated papers published until June 2022.

## 8. Future Perspectives

Different studies revealed altered miRNA expression levels in BD, suggesting that the synergic effects of different miRNAs could contribute to the pathogenesis of the disease. MiRNAs appear to be tightly regulated in adult human brains, with little inter-individual variability. Implementing the most sensitive and precise technical methodologies could be crucial in the precise detection of different expressions among sample groups. Future studies with other cohorts are needed to replicate current findings and determine whether miRNA alterations in BD are widespread throughout the brain or restricted to specific brain regions [65]. It is clear that any future application of plasma miRNA detection for diagnostic or prognostic purposes will depend on the reproducibility and reliability of the results. This could be helpful in the identification of potential BD patients and of the effective targets, which could represent a cure even for those who are refractory to conventional treatment [78].

## 9. Conclusions

This review summarizes the present knowledge of microRNAs involved in BD and their expression and function, together with neural stem cells and their correlation with the altered neuroplasticity observed in BD. Future studies with large cohorts are needed to identify the genes and metabolic pathways regulated by these miRNAs to provide new information and to elucidate the pathogenesis of this disease. We propose several cellular mechanisms involving lithium, miR-134, miR-144, miR-34, and neural stem cells as novel and promising diagnostic and therapeutic tools for a personalized clinical setting in bipolar disorder.

## Figures and Tables

**Table 2:** MicroRNAs and response to mood stabilizers in preclinical studies on animal models of bipolar disorder.

miRNAs	Samples	Mood Stabilizers	Expression Variation	Target Pathways	References
let-7b, let-7c, miR- 128a, miR-24a, miR-30c, miR-34a, miR-221, and miR-144	Rat brain tissue	Lithium or valproate	After 4-week treatment: underexpressed and overexpressed	GRM7, synaptic transmission	Zhou et al., 2009 [106]
miR-221, miR-152, miR-15a, miR-155, miR-34a, miR-181c, and miR-494	Lymphoblastoid cell lines	Lithium	After 4 days of treatment: overexpressed andunderexpressed	Five genes (AP2A1, AP2S1, CD2AP, EIF1, and VCL) significantly enrich three gene ontology-defined biological processes, i.e., macromolecular complex assembly, protein complex assembly, and cellular component assembly	Chen et al. (2009) [40]
miR-34a, miR-495, miR-182, miR-147, and miR-222	Cerebellar granule cell cultures (CGCs)	Lithium Valproate	Downregulated Overexpressed	miR-34a: inhibits the neuroprotective protein Bcl-2 miR-147: downregulates endogenous amyloid precursor protein (APP) expression miR-495: modulates brain-derived neurotrophic factor (BDNF)	Hunsberger et al., 2013 [108]
miR-144	Rat hippocampus	Lithium	Upregulated	Protein kinase C (PKC), Wnt/β-catenin, and PTEN pathways. MiR-144 can inhibit the expression of ataxin 1 involved in mental disorders	Zhou et al., 2009 [106]
miR-134	Plasma	Lithium	Overexpressed	Limk1, controls synaptic development, maturation, and/or plasticity	Rong et al., 2011 [93]
miR-34a	Rat primary neuronal cultures and human SH-SY5Y cells Hippocampus and rodent cerebellar granule cell cultures	Lithium Valproate	Downregulated	Increases the expression of target genes (BCL2, NRF2, and BDNF), producing a neuroprotective effect	Hunsberger et al., 2013 [108]; Wang et al., 2015 [91] Zhou et al., 2009 [106] Alural et al., 2017 [78]

## Data Availability

Not applicable.
